# A novel Aaproach to increase cell wall saccharification for efficient biofuel production

**DOI:** 10.1186/1753-6561-5-S7-P104

**Published:** 2011-09-13

**Authors:** Miron Abramson, Oded Shoseyov, Ziv Shani

**Affiliations:** 1 The Robert H. Smith Institute of Plant Sciences and Genetics in Agriculture, Rehovot, Israel; Futuragene Ltd., Rehovot, Israel; 2 The Robert H. Smith Institute of Plant Sciences and Genetics in Agriculture, Rehovot, Israel; 3 Futuragene Ltd., Rehovot, Israel

## 

The conversion of biomass to biofuels requires heat, pressure and acid treatments to overcome the recalcitrance of the cell wall and remove lignin that hinders access to cellulose. Economic production of biofuels from lignocellulosic biomass and efficient sugar release depends on the ability to reverse and overcome this natural recalcitrance of the plant cell wall.

Most of the current ideas and methods to generate lignocellulose feedstock which is more amenable to saccharification, are limited to the modification or down-regulation of the lignin biosynthesis. This often results in dwarfing, xylem vessel collapse or reduced fitness to stress. In order to overcome these limitations, we present here a novel approach for cell wall remodeling. By introducing new trans-genes from algae, fungi and viruses we create **"Trojan Horses":** polymer pockets of enhanced solubility within the cell wall, thus increasing cellulose accessibility to solvents and enzymes. Two different methodologies are presented. The first includes transgenic introduction of soluble polysaccharides such as **hyaluronan** into the cell wall during its development, leading to its intercalation with the cellulose microfibrils. Upon processing, these soluble intercalated polysaccharides are washed away, resulting in highly porous cell walls with increased accessibility to hydrolytic enzymes. The second methodology involves modification of the cellulose by expression of recombinant **cellobiose dehydrogenase (CDH)** in plant cell walls, leading to disruption of the cellulose microcrystalline lattice and thus increasing hydrolytic enzymes accessibility. Both methodologies result in transgenic plants that exhibit **significantly enhanced cellulose hydrolysis**, while maintaining their physiological and structural integrity.

This novel concept provides new prospects for more economical production of liquid biofuel to substitute fossil fuels.

